# Highly Sensitive NO_2_ Gas Sensors Based on MoS_2_@MoO_3_ Magnetic Heterostructure

**DOI:** 10.3390/nano12081303

**Published:** 2022-04-11

**Authors:** Wei Li, Mahboobeh Shahbazi, Kaijian Xing, Tuquabo Tesfamichael, Nunzio Motta, Dong-Chen Qi

**Affiliations:** 1 School of Chemistry and Physics, Queensland University of Technology, Brisbane, QLD 4001, Australia; w73.li@hdr.qut.edu.au (W.L.); mahboobeh.shahbazi@qut.edu.au (M.S.); 2 Centre for Materials Science, Queensland University of Technology, Brisbane, QLD 4001, Australia; 3 School of Physics and Astronomy, Monash University, Clayton, VIC 3800, Australia; kaijian.xing@monash.edu; 4 School of Mechanical, Medical and Process Engineering, Queensland University of Technology, Brisbane, QLD 4001, Australia

**Keywords:** heterostructure, 2D materials, sulfurization, nitrogen dioxide, gas sensor, molybdenum trioxide, molybdenum disulfides

## Abstract

Recently, two-dimensional (2D) materials and their heterostructures have attracted considerable attention in gas sensing applications. In this work, we synthesized 2D MoS_2_@MoO_3_ heterostructures through post-sulfurization of α-MoO_3_ nanoribbons grown via vapor phase transport (VPT) and demonstrated highly sensitive NO_2_ gas sensors based on the hybrid heterostructures. The morphological, structural, and compositional properties of the MoS_2_@MoO_3_ hybrids were studied by a combination of advanced characterization techniques revealing a core-shell structure with the coexistence of 2H-MoS_2_ multilayers and intermediate molybdenum oxysulfides on the surface of α-MoO_3_. The MoS_2_@MoO_3_ hybrids also exhibit room-temperature ferromagnetism, revealed by vibrating sample magnetometry (VSM), as a result of the sulfurization process. The MoS_2_@MoO_3_ gas sensors display a *p*-type-like response towards NO_2_ with a detection limit of 0.15 ppm at a working temperature of 125 °C, as well as superb selectivity and reversibility. This *p*-type-like sensing behavior is attributed to the heterointerface of MoS_2_-MoO_3_ where interfacial charge transfer leads to a *p*-type inversion layer in MoS_2_, and is enhanced by magnetic dipole interactions between the paramagnetic NO_2_ and the ferromagnetic sensing layer. Our study demonstrates the promising application of 2D molybdenum hybrid compounds in gas sensing applications with a unique combination of electronic and magnetic properties.

## 1. Introduction

The rapid development in technologies and industrial activities in recent decades has brought unprecedented convenience to our life. However, it has also led to a rapid increase in the emission of toxic and greenhouse gases bringing harm to our planet and human health [1,2]. Toxic gases, such as CO, NO_2_, SO_2_, NO, NH_3_, etc., inflict respiratory damage on humans, such as lung cancers, cough, and asthma, even at an extremely low concentration [2]. Among them, NO_2_ acting as a typical toxic gas has received particular attention in the last decades. NO_2_ not only produces acid rain harming the environment but is also a major contributor to severe respiratory symptoms when its concentration is higher than 1 ppm [3,4,5,6]. As a consequence, it is crucially necessary to develop highly selective, sensitive, rapid response, and low-cost sensors for detecting and monitoring trace amounts of NO_2_ [7].

To meet this challenge, numerous semiconducting materials have been extensively studied to develop conductometric gas sensors which are capable of detecting a wide range of analytical gases [8], such as metal oxide semiconductors, conducting polymers, carbon nanotubes, and metal dichalcogenide semiconductors. Among these sensitive materials, metal oxides semiconductors (MOSs) have attracted the most attention for utilization in the preparation of gas sensors due to their simple structure, high sensitivity, chemical stability, and low cost. Particularly, MOSs have been developed in great numbers of nanostructures with different dimensions, such as one-dimensional (1D) nanoribbon, nanowire, nanorod, two-dimensional (2D) nanosheet, nanoplate, nanoflake, and three-dimensional (3D) nanoflower, the nanoarray [5,7]. The nanostructured MOSs have a high surface-to-volume ratio which supports more adsorption sites for analyte gas molecules to enhance the gas sensing behavior. However, the high working temperature, low selectivity, and high power consumption also impede their wider and more versatile applications [9,10].

Recently, two-dimensional (2D) materials have emerged as a new materials platform enabling breakthroughs in fundamental research and transformative technologies [11,12]. The absence of surface dangling bonds and unique atomic-level uniformity make them very appealing for developing a plethora of optical, electronic, and energy applications. 2D materials also have intrinsic high surface-to-volume ratio providing abundant adsorption sites, as well as enhanced electronic properties due to carrier confinement. These distinct attributes make 2D materials particularly attractive for gas sensing applications [7,13,14,15]. Indeed, gas sensors based on typical 2D materials including graphene, MXenes, transition metal dichalcogenides (TMDCs), and phosphorene have been widely demonstrated [15,16,17]. In particular, MoS_2_, predicated theoretically as one of the most prominent candidates for 2D material-based gas sensors [18,19], has yielded a promising sensing performance to NO_2_ albeit while still presenting challenges, such as low sensitivity and unsatisfactory selectivity [20], which limit practical applications [20,21].

On the other hand, layered metal oxides have also emerged as a new class of van der Waals 2D materials [22]. With the combination of the desirable properties of both oxide materials and 2D materials, they exhibit great potential in numerous fields, such as gas sensing, electronics, optics, catalysis, and energy storage [23,24,25,26,27]. The thermodynamically stable α-MoO_3_ is a typical 2D oxide with a layered crystal structure and has received a lot of attention recently for building novel electronic and optoelectronic devices [28]. It has also been demonstrated for various gas sensing applications [29,30], including achieving ultrahigh sensitivity and superior selectivity to NO_2_ [27,31]. Driven by the prominent prospect of 2D van der Waals heterostructures with rationally tailored properties [32,33], enormous efforts have also been devoted to the synthesis and study of hybrid compounds of MoS_2_@MoO_3_. For instance, electrocatalysts based on MoO_3_@MoS_2_ nanosheets were reported to enhance hydrogen evolution reaction [24,34,35,36], and MoO_3_@MoS_2_ nanowires were also reported as anodes in lithium-ion batteries [37]. There has also been increasing interest in MoS_2_@MoO_3_ composites in gas sensing. Singh et al. synthesized MoS_2_@MoO_3_ nanocomposites with the hydrothermal method for NH_3_ sensing in a humid environment [38]; Li et al. showed an enhanced CO gas sensor based on MoS_2_@MoO_3−x_ nanosheets synthesized via the solvothermal method [39]. However, NO_2_ gas sensors based on MoS_2_@MoO_3_ heterostructure remain largely unexplored.

In this work, we developed a highly sensitive NO_2_ sensor based on MoS_2_@MoO_3_ nanoribbons by a post-sulfurization method. The morphology and structures of the as-synthesized MoS_2_@MoO_3_ hybrid heterostructures were characterized by field-emission scanning electron microscopy (FE-SEM), and transmission electron microscopy (TEM). The structural, compositional, and magnetic properties of the MoS_2_@MoO_3_ hybrids were fully investigated by X-ray diffraction (XRD), Raman spectroscopy, X-ray photoelectron spectroscopy (XPS), and magnetic measurement. The MoS_2_@MoO_3_ nanoribbons-based gas sensor exhibited a *p*-type gas sensing behavior to the analytical gases, with high sensitivity, impressive selectivity, and good reproducibility to NO_2_ at an optimal operating temperature of 125 °C.

## 2. Materials and Methods

### 2.1. Synthesis of MoO_3_

**Figure 1:**
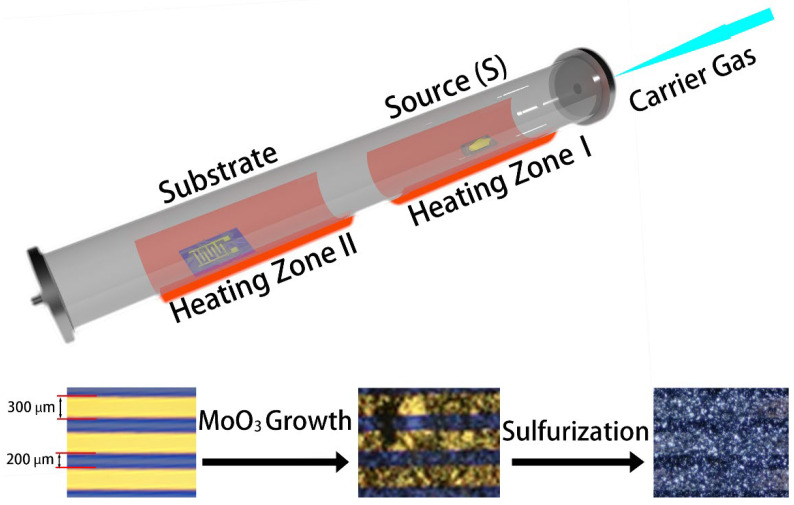
**Above**: schematic illustration of the post-sulfurization process for MoO_3_ nanoribbons, the SiO_2_/Si substrate with pre-patterned interdigital electrodes. **Below**: the optical images of the device before and after MoO_3_ synthesis and subsequent sulfurization.

Before the deposition of MoO_3_ nanoribbons, Au/Ti (100 nm/10 nm) interdigital contact electrodes were deposited on SiO_2_ (285 nm)/Si substrates through a shadow mask by using electron-beam physical vapor deposition (E-beam PVD). The interdigital electrodes consist of four pairs of electrodes with a width of 300 µm and a spacing of 200 µm (Figure 1). MoO_3_ nanoribbons were then synthesized via vapor phase transport (VPT) directly on the substrates with pre-patterned electrodes, following our previous reported approach (Figure 1) [27]. In brief, 0.125 g MoO_3_ (99.95% purity, Strem Chemicals) powder was placed in the center of high-temperature heating Zone I (upstream of the tube) of a dual-zone furnace (LABEC—Split Horizontal Tube Furnace) inside a quartz tube (1000 mm in length, 50 mm in diameter), where the temperature was set to 750 °C (Figure 1). Simultaneously, the SiO_2_/Si substrate was placed in the low-temperature Zone II (downstream of the tube) with a temperature of 600 °C. Synthetic air (hydrocarbon impurities < 0.1 ppm) was used as the carrier gas with an airflow of 120 sccm.

### 2.2. Sulfurization

The subsequent sulfurization of the synthesized MoO_3_ nanoribbons on SiO_2_/Si substrates was performed in the same quartz tube by replacing the boat with MoO_3_ powder with 0.6 g sulfur powder (99.3% purity, Ajax Finechem, Sidney, NSW, Australia) at a temperature of 280°. Meanwhile, the substrates with MoO_3_ nanoribbons were placed in the same location with a temperature of 550°. Pure argon was used as the carrier gas with an airflow of 120 sccm.

### 2.3. Materials Characterizations

A Leica DM6000 optical microscope (Weltzar, Germany) and Zeiss sigma field emission scanning electron microscope (FESEM, Jena, Germany) were utilized to characterize the surface morphology of the as-grown samples before and after sulfurization. Transmission electron microscopy (TEM) and energy dispersive spectroscopy (EDS) were collected using a JEOL 2100 electron microscope (Tokyo, Japan) to analyze the detailed structure and crystallinity of the as-grown samples after sulfurization. The X-ray diffraction (XRD) pattern was carried out using PANaytical MPD Cu powder XRD with Cu *K*_α_ radiation source (Almelo, Netherlands) to study the phase compositions and crystallographic structures of the as-synthesized samples before and after sulfurization. A Raman microscope (Renishaw inVia, Wotton-under-Edge, UK) with an excitation laser of 532 nm in wavelength was employed to study the structural vibration of the samples both before and after sulfurization. The stoichiometry and electronic structure of as-grown samples after sulfurization were studied by X-ray photoelectron spectroscopy (XPS) with a Kratos AXIS Supra photoelectron spectrometer using a monochromatized Al *K*_α_ X-ray source (Manchester, UK). The magnetic property of the sulfurated samples was measured by a vibrating-sample magnetometer (VSM) using a Cryogen-free 5 T system (London, UK).

### 2.4. Characterization of Gas Sensors

A home-built multichannel gas sensing test system was used to evaluate the gas sensing performance of the fabricated gas sensor, shown in Figure S2. The gas sensor was mounted at the top of the ceramic heater providing on operating temperature for the device controlled by an Agilent E3649 power supply (Santa Clara, CA, USA). The test chamber with an internal volume of 1100 mL was coupled to five high precision mass flow controllers (MFC, MKS 1479A, 200 sccm, Andover, MA, USA) capable of supporting five different analytical gases, which were controlled and mixed by a vacuum/mass system controller (MKS 946, Andover, MA, USA). The ratio of flow rates of analytical gases and the dry synthetic air were adjusted by the relative MFC while keeping a constant total flow of 200 sccm inputing to the chamber to obtain the desired concentration of analytical gas for the gas sensing measurements. The conductometric sensing performance of the gas sensor was evaluated by measuring the resistance of the gas sensor exposed in analytical gases at a bias voltage of 1 V by a Keithley 6487 sourcemeter unit (Solon, OH, USA). The sensing response *R* of the gas sensor to analytical gas is defined as:

(1) $$R\;\left(\%\right)=\frac{R_{g}-R_{a}}{R_{g}}\times 100$$

where *R_g_* and *R_a_* are the resistance of the sensor exposed in analytical gas and synthetic air, respectively. The polarity of *R* is hence directly related with the direction of resistance change upon exposure to analytical gas.

## 3. Results and Discussion

### 3.1. Materials Characterization

The sulfurization of MoO_3_ in this work was carried out inside the twin-zone tube furnace as described in the Materials and Methods section by exposing as-grown MoO_3_ to sulfur vapor. The overall reaction of MoO_3_ with sulfur vapor during the sulfurization process could be described as [40,41,42,43,44]:

(2) $$2MoO_{3}+7S\to 2MoS_{2}+3SO_{2}$$

In this reaction, sulfur atoms progressively remove oxygen from MoO_3_ by forming gaseous SO_2_ whilst bonding with Mo to form MoS_2_. The relative stoichiometric ratio of S to Mo at the substrate surface hence plays a significant role that will directly impact the composites of the product. Incomplete sulfurization can lead to the formation of intermediate molybdenum oxysulfides (MoO_3−x_S_y_; 0 ≤x ≤3;0 ≤y ≤2) described by the following reaction pathway [40]:

(3) $$2MoO_{3}+\left(2y+x\right)S\to 2MoO_{3-x}S_{y}+xSO_{2}$$

**Figure 2:**
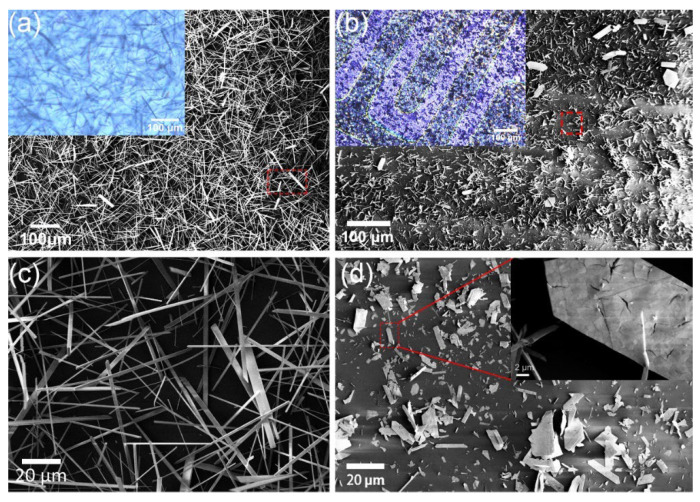
SEM image of (**a**) the as-grown α-MoO_3_ nanoribbons network on SiO_2_/Si interdigitated substrate, and (**b**) the sulfurized α-MoO_3_ nanoribbons. Insets in (**a**,**b**) shows the corresponding optical micrographs. The high-resolution SEM image of (**c**) the as-grown α-MoO_3_ and (**d**) the sulfurized α-MoO_3_. The inset in (**d**) is a magnified view of an individual sulfurized MoO_3_ nanoribbon.

Optical microscopy and SEM were employed to characterize the evolution of morphologies of the as-prepared α-MoO_3_ nanoribbons before and after sulfurization. As shown in Figure 2a, the as-grown α-MoO_3_ samples grew randomly on the substrate with a high density and possessed a rectangular shape with a typical length of 100 μm and a width of 5 μm (Figure 2c). The morphology of the α-MoO_3_ nanoribbons is consistent with those reported earlier using the same synthesis condition [27]. The post-sulfurization directly proceeded on the surface of the as-grown α-MoO_3_ nanoribbons in the sulfur vapor environment. After sulfurization, the samples changed in a few aspects. Firstly, the color of the nanoribbons changed from colorless (i.e., transparent) to dark violet (c.f. optical micrographs in Figure 2a,b), indicating a reduction in the optical bandgap and consistent with the color change expected for the sulfurization of MoO_3_ [45]. Meanwhile, the density of nanoribbons on the substrate noticeably declined, suggesting the evaporation of MoO_3_ that consumes the as-grown MoO_3_ nanoribbons. Secondly, as a result of the sulfurization, the shape of individual nanoribbons changed from elongated rectangles (Figure 2c) to irregular shapes (Figure 2d) and the edges of the crystals appeared to be less well-defined; the surface of the nanoribbons also became rougher with the presence of cracks suggesting that sulfurization alters the crystal structure of MoO_3_ on the surface leading to lattice mismatch among the hybrid molybdenum compounds. The morphological changes of the sulfurized MoO_3_ are most likely brought through a complex combination of solid-gas reaction, atomic interdiffusion, evaporation, and deposition [46]. Furthermore, the lateral size of crystals reduced significantly to about 20 μm in length from the original length of about 100 μm.

**Figure 3:**
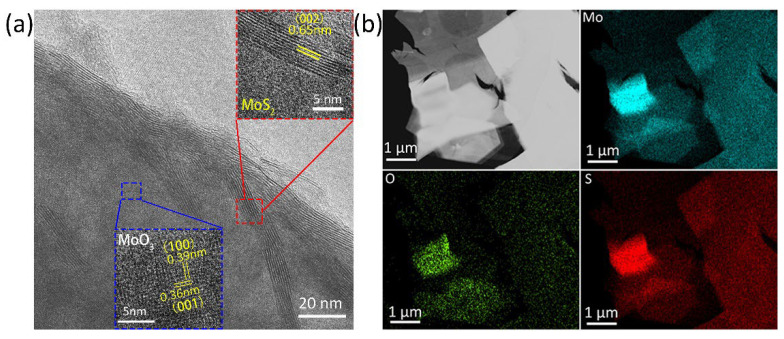
(**a**) TEM image and (**b**) EDS element mapping of the sulfurized MoO_3_ nanoribbons.

High-resolution TEM images were collected to further study the structural crystallinity of the sulfurated MoO_3_ nanoribbons. To prepare TEM samples, substrates with sulfurized MoO_3_ nanoribbons were placed in ethanol to obtain a well-dispersed solution, after which droplets of the as-prepared solution were drop-casted on lacey-supported 300 mesh copper grids. Figure 3a shows a representative high-resolution transmission electron microscopy (HRTEM) image taken from the edge of sulfurized MoO_3_. The lattice spacing of 0.36 nm and 0.39 nm corresponds nicely to the (001) and (100) plane of α-MoO_3_, whereas the lattice spacing of 0.65 nm of layers at the edge of the flake is consistent with the (002) plane of 2H-MoS_2_, suggesting that the outer layers of α-MoO_3_ have been partly converted to MoS_2_ through the sulfurization process. The sulfurization of MoO_3_ has been further confirmed by the EDS element mapping in Figure 3b which shows an even distribution of S in addition to Mo and O elements.

**Figure 4:**
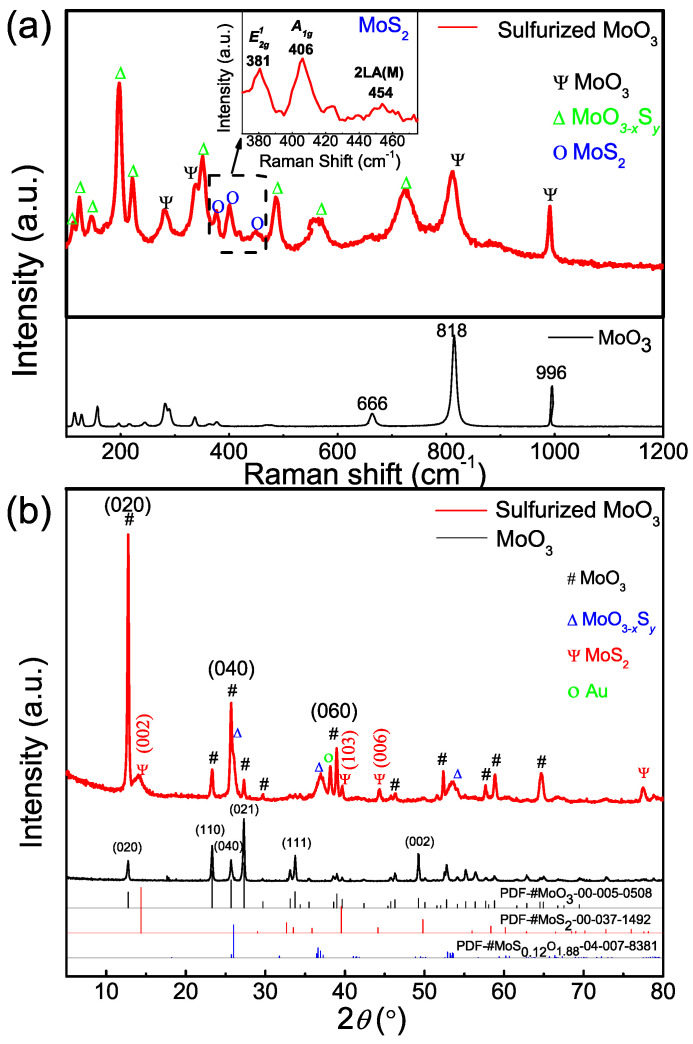
(**a**) Raman spectra and (**b**) XRD patterns of MoO_3_ on SiO_2_/Si substrate before and after sulfurization.

Raman spectra were also collected to further understand the structural change of MoO_3_ by sulfurization. As shown in Figure 4a, the Raman spectrum for as-grown MoO_3_ (black line) exhibits the expected Raman modes for α-MoO_3_ with a layered structure [47,48]. In particular, it has well-defined Raman features associated with the vibration modes of the oxygen atoms terminating the van der Waals layer at 996 cm^−1^ (M = O asymmetrical stretching), as well as the stretching mode of the O-Mo bonds at 818 cm^−1^ for the corner-shared oxygen and at 666 cm^−1^ for the edge-shared, triply bonded oxygen [49,50]. After sulfurization, the Raman spectrum (red line) displays new peaks in addition to retaining the majority of the characteristic features of MoO_3_. Most notably, the new peaks at 381 cm^−1^ and 406 cm^−1^ are consistent with the characteristic in-plane $E_{2g}^{1}$ vibration mode and the out-of-plane $A_{1g}$ vibration mode of 2H-MoS_2_, respectively [46,51]. The two Raman peaks have a frequency difference of ~25 cm^−1^, suggesting that the MoS_2_ on the surface of MoO_3_ is multilayer, which is consistent with the HRTEM results. Moreover, the peak 454 cm^−1^ is assigned to the second order 2LA(M) phonons of MoS_2_ which are generally present in multilayers of MoS_2_ [52,53]. There are, however, additional Raman peaks that cannot be exclusively assigned to MoO_3_ or MoS_2_, such as strong Raman features at 198, 222, 560, and 730 cm^−1^, and they are best assigned to the vibration modes of Mo-O and Mo-S bonds in intermediate compound molybdenum oxysulfide (MoO_3−x_S_y_) as a result of incomplete sulfurization described in the reaction in Equation (2) [40]. The co-existence of MoO_3_, MoS_2_, and the intermediate molybdenum oxysulfide is further corroborated by XRD. Figure 4b shows the XRD patterns of MoO_3_ before (black line) and after (red line) sulfurization. The intense diffraction peaks of pristine MoO_3_ can all be indexed to the orthorhombic α-MoO_3_ (JCPDS Card No. 00-005-0508) including the (0 2 0) and (0 4 0) planes that are characteristic for layered MoO_3_ [27,54,55]. After sulfurization, the XRD patterns display several additional diffraction peaks including the diffraction peaks with 2θ at 14.4°, 39.5°, and 44.3° corresponding to reflections of the (002), (103), and (006) planes of 2H-MoS_2_ (JCPDS No. 00-037-1492) [44,56], confirming again the formation of MoS_2_ on the MoO_3_ nanoribbons after sulfurization. With the 2θ position of the (002) diffraction peak and the wavelength of Cu K_α_ line (λ = 0.15418 nm), we can arrive at an interlayer distance of 0.62 nm for MoS_2,_ in good agreement with the d-spacing determined by HRTEM in Figure 3a. The formation of intermediate MoO_3−x_S_y_ is also supported by the appearance of additional XRD patterns consistent with those from a representative molybdenum oxysulfide MoS_0.12_O_1.88_ (JCPDS No. 04-007-8381).

**Figure 5:**
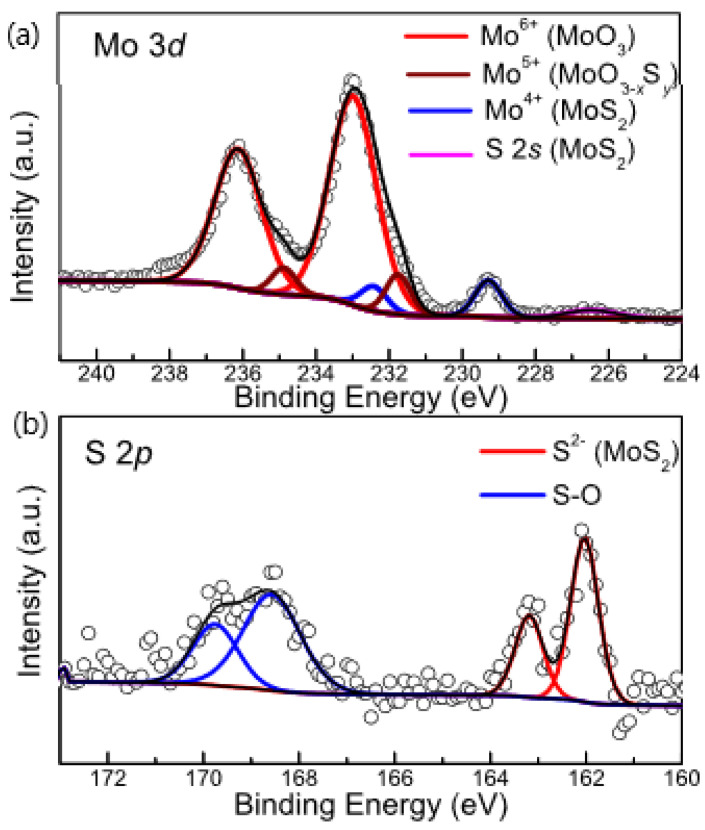
XPS spectra of (**a**) Mo 3*d* core-levels and (**b**) S 2*p* core-levels of sulfurized MoO_3_ on SiO_2_/Si substrate.

X-ray photoelectron spectroscopy (XPS) characterization was also carried out to determine the compositions and valence states of sulfurized MoO_3_. The binding energy of XPS spectra was calibrated using adventitious carbon (284.8 eV) as a reference. As shown in the wide survey scan in Figure S1, no other unexpected contaminants were introduced after sulfurization. Figure 5a shows the Mo 3*d* core-level spectrum which can be deconvoluted to three distinct 3d spin-orbit split doublets fitted by a Voigt line shape. The XPS doublets dominating the high binding energy part of the spectrum at 236.1 eV (Mo 3d_3/2_) and 233.0 eV (Mo 3*d*_5/2_) are assigned to the characteristic Mo^6+^ state in α-MoO_3_ [57]. Meanwhile, the Mo 3d doublets positioned at 232.4 and 229.3 eV are consistent with Mo^4+^ in 2H-MoS_2_, with the accompanied S 2s peak at 226.5 eV [58,59]. The Mo 3*d* doublets at 234.9 eV (Mo 3*d*_3/2_) and 231.8 eV (Mo 3*d*_5/2_), located in between those of the Mo^6+^ and Mo^4+^ components, can be ascribed to Mo in MoO_3−x_S_y_ with a nominal oxidation state of 5^+^ [24,60]. Correspondingly, S 2*p* core-level spectrum in Figure 5b shows the S 2*p* doublets at 163.2 eV (S 2*p*_1/2_) and 162.1 eV (S 2*p*_3/2_) originating from Mo-S bonds in 2H-MoS_2_ [35,36,42]. In addition, the peaks at 169.7 eV (S 2*p*_1/2_) and 168.6 eV (S 2*p*_3/2_) correspond to the S-O bonds both in the intermediate molybdenum oxysulfide and potentially the adsorbed SO_2_ on the surface of MoO_3_ as a result of the sulfurization reaction [51,61,62].

**Figure 6:**
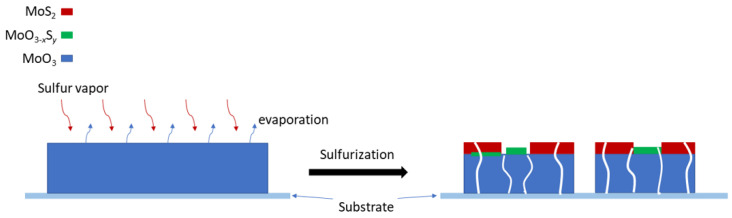
A schematic diagram illustrating the sulfurization process of MoO_3_ nanoribbons.

The above structural and compositional analysis shows that the sulfurization process induces the formation of MoO_3_/MoS_2_ hybrid core-shell structure with the co-existence of incompletely sulfurized oxysulfide intermediates embedded on the surface, as shown schematically in Figure 6. Because of the relatively high temperature of the sulfurization process, the reaction also involves evaporation of MoO_3_ and re-deposition which consume the initial MoO_3_ nanoribbons, giving rise to the reduced lateral dimensions and morphological changes of sulfurized MoO_3_ as discussed above_._ Although the overall molar ratio of S:MoO_3_ for the respective sources exceeds the required ratio of 7:2 as in reaction Equation (1) for complete sulfurization, at regions where the high-density MoO_3_ nanoribbons overlap inhibiting the flow of S vapor to react completely with the outer layers of MoO_3_, the local effective ratio could be lower leading to the formation of incompletely sulfurized oxysulfide intermediates.

**Figure 7:**
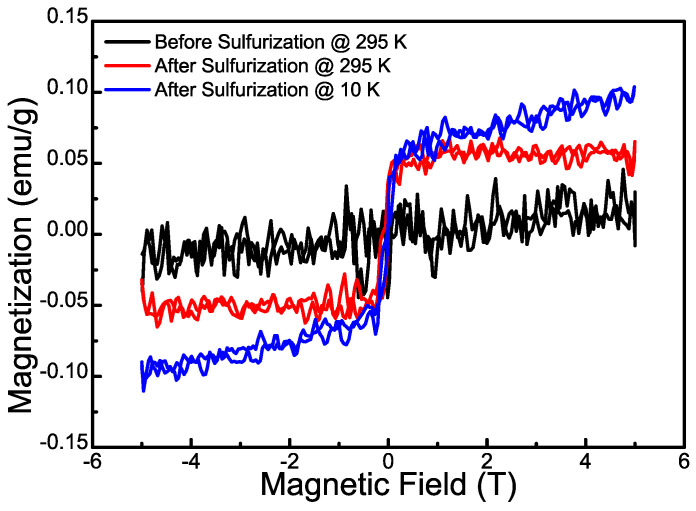
Magnetic hysteresis loop of pristine MoO_3_ and sulfurized MoO_3_ at different temperatures.

Because NO_2_ carries a magnetic moment originating from its unpaired valence electron, the magnetic properties of sensing layers could potentially benefit gas sensing performance by enhancing affinity to substrates through magnetic dipole interactions [63]. Although both α-MoO_3_ and MoS_2_ are intrinsically non-magnetic, ferromagnetism could be introduced through defects, edges, and non-stoichiometry that induce unpaired electrons in Mo 4d orbitals [64,65,66,67,68]. We therefore measured the magnetic properties of the sulfurated MoO_3_ samples by VSM with an applied magnetic field in the range of −5 T to 5 T. Figure 7 presents a comparison of the magnetization vs. magnetic field for sulfurized MoO_3_ samples at room temperature and 10 K and the pristine MoO_3_. The pristine MoO_3_ nanoribbons do not exhibit any hysteretic behaviors, consistent with its non-magnetic character with a Mo 4d^0^ configuration. After sulfurization, the MoS_2_@MoO_3_ sample clearly shows ferromagnetic behavior with a specific saturation magnetization (M_s_) about 0.05 emu/g at room temperature. The saturation magnetization at 10 K is comparable with that of room temperature, but it further increases slightly with at large magnetic field and did not saturate up to 5 T. This magnetization behavior suggests a combination of ferromagnetic and paramagnetic properties. The observed ferromagnetism is comparable to those reported for defective MoS_2_ and MoO_3_ with defect-induced ferromagnetism [64,65,69], and therefore could also originate from the prevalent defects as a result of the sulfurization process. In addition, the edges of MoS_2_ have been shown to display robust ferromagnetism [70]; the MoS_2_ nanosheets that cover the surface of MoO_3_ are expected to give rise to plenty of edge states that enhance the ferromagnetism. Furthermore, the existence of Mo^5+^ with an unpaired 4d electron (4d^1^ configuration), as revealed in Figure 5a by XPS, could also provide magnetic moments for the sulfurized MoS_2_@MoO_3_ hybrid structures [65]. Lastly, interface-induced ferromagnetism due to interfacial charge transfer [66] could also occur at the MoS_2_@MoO_3_ heterointerface by introducing spin magnetic moments into the Mo-4d conduction bands of MoO_3_. It is worth noting that some of the magnetic moments introduced by sulfurization could be isolated lacking long-range ferromagnetic coupling, giving rise to the observed paramagnetic behavior as mentioned above.

### 3.2. Gas-Sensing Performance

**Figure 8:**
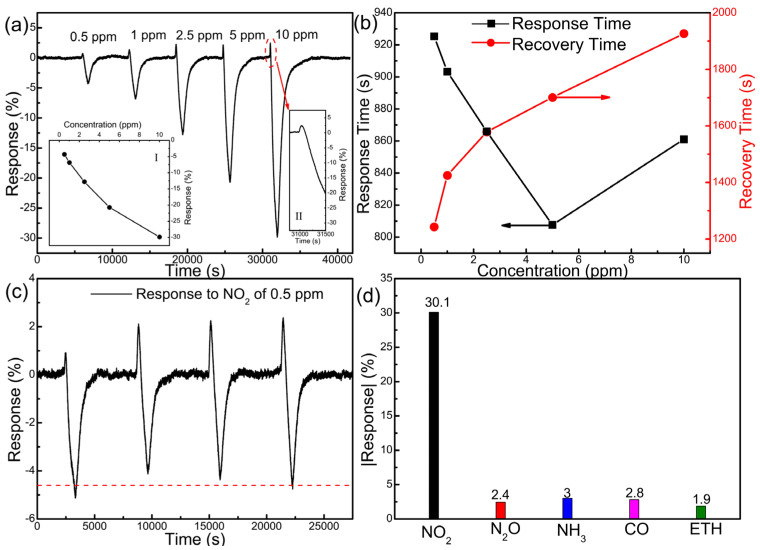
(**a**) Dynamic sensing response of the MoS_2_@MoO_3_ based sensor to NO_2_ at concentrations ranging from 0.5 ppm to 10 ppm under the working temperature of 125 °C. Inset I is a plot of the sensing response as a function of NO_2_ concentration and inset II is the enlarged view of the initial positive response to 10 ppm NO_2_. (**b**) Response and recovery time of the MoS_2_@MoO_3_-based sensor toward NO_2_ from 0.5 ppm to 10 ppm at the working temperature of 125 °C. (**c**) Cyclic response of the sensor toward 0.5 ppm NO_2_. (**d**) Selectivity of the sensor in absolute response to NO_2_, N_2_O, NH_3_, CO, and ETH of 10 ppm concentration. Measurements were performed at the optimal operating temperature of 125 °C.

The gas sensor was fabricated by depositing electrodes on SiO_2_/Si substrates before α-MoO_3_ synthesis and the subsequent sulfurization. The gas-sensing performance for NO_2_, including the dynamic sensing response, response and recovery time, reversibility, and selectivity of the sulfurized MoO_3_ gas sensors was evaluated with the multichannel gas sensing system (see Figure S2 for the schematics of the sensing test system). All the data for the gas sensing performance were collected at the optimal operating temperature of 125 °C, which was determined by measuring the sensing response as a function of the working temperature of the sensors which was controlled via the ceramic heater underneath. In contrast to positive responses (i.e., increase of resistance) upon exposure to NO_2_ (typical oxidizing gas) usually displayed by metal oxide conductometric gas sensors including α-MoO_3_ [10,27,71], the sulfurized device displays a *p*-type response (i.e., decrease of resistance shown in Figure S3) to NO_2_ with a concentration in the range of 0.5 ppm to 10 ppm (Figure 8a). The device presents a maximum negative response of −30.1% to NO_2_ at 10 ppm and a small but noticeable response of −4.3% to 0.5 ppm of NO_2_. The dependence of response on NO_2_ concentration is displayed in the inset of Figure 8a which deviates from a linear dependence at high concentration, suggesting the adsorption of NO_2_ on the surface of sulfurized MoO_3_ starts to be limited at high concentration. According to the dependence at the low concentrations (inset I of Figure 8a), we can also determine a limit of detection (LOD) of ~0.15 ppm by implementing the IUPAC method [72]. Intriguingly, the sensors display a small positive response upon the input of NO_2_ before the dominating negative response (inset II of Figure 8a), the origin of which will be discussed below. Figure 8b illustrates the response time (τ_res_) and recovery time (τ_rec_) of the device based on MoS_2_@MoO_3_ towards NO_2_ from 0.5 ppm to 10 ppm at the optimal temperature of 125 °C (also see Figure S4 of Supplementary Material for a detailed presentation of response and recovery time to 10 ppm NO_2_). In general, the response time decreases with increasing concentration of NO_2_ (<5 ppm) whereas the recovery time exhibits an opposite trend. Similar dependence has been reported before and is explained with the Freundlich law which dictates a higher diffusion and reaction process with NO_2_ molecules adsorption (hence faster response dynamics) on the surface at a higher concentration [73,74]. The corresponding desorption is also going to take longer at higher concentration because of the higher number of adsorbed molecules. Intriguingly, the response time at the highest concentration of 10 ppm experiences an increase. It is not exactly clear what causes this anomaly that deviates from the Freundlich law. One possible cause is the initial positive response spike following gas introduction that goes into the calculation of response time (Figure S4). At high NO_2_ concentration, the positive response spike also increases in magnitude and may cause the negative response to take longer to reach its maximum value. It is worth noting that the nominal response and recovery time of our sensors tend to be overestimated due to the set-up of the sensing test chamber with a large internal volume of 1100 mL. It therefore requires a significantly extended duration to completely replace this volume with the analytical gas at the desired concentration and to purge it away to return to the baseline [27]. However, their dependence on gas concentration (as presented in Figure 8b) is still relevant. The reproducibility and reversibility of the gas sensors are also presented in Figure 8c, in which the device operated with NO_2_ at 0.5 ppm in four consecutive cycles over an 8 h testing period. The sensors show a reproducible response with a standard deviation of response less than 0.4%. The long-term stability of this sensor was also measured for 8 weeks (Figure S5), in which the response to NO_2_ decreased from −30.1% to −23% in the first week and then it kept a relatively stable response about −22% in 7 weeks. Lastly, the selectivity of the sulfurized MoO_3_ sensors was studied by measuring responses to a few interference gases including N_2_O, NH_3_, CO, and ethanol (ETH) as shown in Figure 8d. All the responses to the analytical gases were collected at 10 ppm concentration. The MoS_2_@MoO_3_ gas sensors clearly exhibit a good selectivity to NO_2_; we attribute the selectivity to the enhanced magnetic interaction between the paramagnetic NO_2_ and the ferromagnetic MoS_2_@MoO_3_ sensing layer. Upon NO_2_ adsorption, it induces additional magnetic dipole interaction with the substrate in addition to a surface electric dipole (due to the polar character of NO_2_), giving rise to a stronger adsorption affinity than the other non-magnetic interference gas molecules.

### 3.3. Gas-Sensing Mechanism

**Figure 9:**
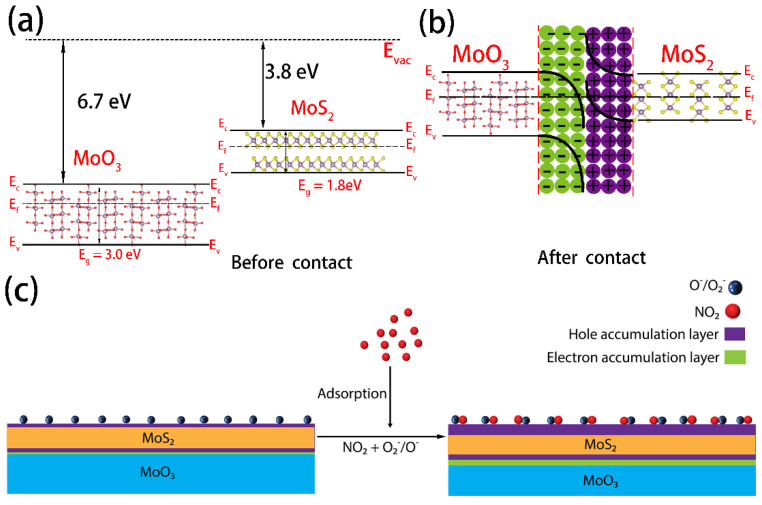
The energy band diagram of MoS_2_@MoO_3_ heterostructure (**a**) before contact, (**b**) after contact; (**c**) gas-sensing mechanism of MoS_2_@MoO_3_ heterostructure.

Both α-MoO_3_ and MoS_2_ are typical n-type semiconductors due to the prevalence of anion vacancies (i.e., O and S vacancies) in their pristine structure due to their low formation energy [75,76]. Anion vacancies act as shallow donors in the bandgap leading to n-type conduction behavior. It is well accepted that n-type semiconductors usually respond to oxidizing gas such as NO_2_ with an increase in resistance (i.e., positive response) as a result of electron depletion in the sensing materials following electron transfer to analytical gas molecules facilitated through the surface adsorbed oxygen ions [77]. The reversal of the response polarity (i.e., decrease in resistance) to NO_2_ in our MoS_2_@MoO_3_ hybrid sensors is therefore intriguing. Due to an extremely high electron affinity of 6.7 eV [75,78], MoO_3_ is a potent electron acceptor and has been widely used as an interfacial layer to induce or enhance *p*-type conduction in a wide range of electronic materials including organic semiconductors, diamond, graphene, and TMDCs through the charge transfer doping process [79]. We therefore attribute the negative sensing response to the heterointerface formed between MoO_3_ and MoS_2_ with the concomitant interfacial charge exchange. As illustrated in the schematic energy band diagram in Figure 9, MoS_2_ has an ionization energy of ~5.6 eV [80], and it therefore forms a type-III broken-gap heterojunction with MoO_3_ with a much deeper conduction band. Because of this extreme band offset, electrons flow spontaneously from the valance band of MoS_2_ to the conduction band of MoO_3_ until the Fermi level is equated at thermodynamic equilibrium [81], leading to strong band bending across the interface. The interfacial electron transfer not only depletes the initial free electron carriers in MoS_2_ but also moves the Fermi energy further to form a MoS_2_ inversion layer with *p*-type character [82]. When exposed to air, the hole accumulation layer in MoS_2_ is enhanced because of further electron transfer from the MoS_2_ to adsorbed oxygen molecules to form different negative oxygen ion species ($O_{2}^{-}$ and $O^{-}$) [83,84].

(4) $$O_{2}+e^{-}\to O_{2}^{-}$$

(5) $$O_{2}+2e^{-}\to \;2O^{-}\;$$

Upon exposure to NO_2_, the absorbed NO_2_ molecules directly interact with MoS_2_ or through the negative oxygen ion species to capture electrons, accompanied by further hole generating in MoS_2_ [85,86]:

(6) $$NO_{2\left(gas\right)}+e^{-}↔NO_{2\left(ads\right)}^{-}$$

(7) $$NO_{2\left(gas\right)}+O_{2\left(ads\right)}^{-}+2e^{-}\to NO_{2\left(ads\right)}^{-}+2O_{\left(ads\right)}^{-}$$

(8) $$NO_{2\left(gas\right)}+O_{\left(ads\right)}^{-}+2e^{-}\to NO_{2\left(ads\right)}^{-}+2O_{\left(ads\right)}^{2-}$$

In addition, NO_2_ can also interact with S vacancies ($V_{S}^{++}$) at the surface MoS_2_ [84], again generating holes into the valance band [83,85,86,87].

(9) $$NO_{2\left(ads\right)}^{-}+V_{S}^{++}\to NO_{2\left(gas\right)}+h^{+}$$

Consequently, the hole accumulation layer in MoS_2_ is further enlarged with concomitant increase of hole density, which is translated into a decrease of resistance in response to NO_2_.

It is worth noting that the resistance change of the n-type α-MoO_3_ upon NO_2_ adsorption should remain positive and counteract the resistance change of the *p*-type MoS_2_ hole accumulation layer. This may explain the positive response spike immediately following NO_2_ introduction as in Figure 8a. However, since the thickness of MoS_2_ is expected to be much lower than that of MoO_3_ and comparable to the Debye length, its sensing response is maximized to dominate the overall sensing behavior [77], thereby resulting in a net decrease of resistance (i.e., negative response). Similar phenomena have been observed in other materials (e.g., a-RP [88], SnO_2_ [89], and CuO/ZnO [90]).

## 4. Conclusions

In conclusion, we report MoS_2_@MoO_3_ ferromagnetic heterostructure synthesized by post-sulfurization of α-MoO_3_ nanoribbons on SiO_2_/Si substrate. Gas sensors fabricated with the sulfurized MoO_3_ as sensing layers MoS_2_@MoO_3_ display a *p*-type response towards NO_2_ with high sensitivity in the range of 0.5 ppm to 10 ppm with a LOD of ~0.15 ppm at the optimal working temperature of 125 °C. The response and recovery time of the sensors, although sluggish due to the nature of the testing apparatus, generally follows the Freundlich model which governs the gas adsorption and diffusion on the surface. The gas sensor also presents good reversibility and impressive selectivity against interfering gases including N_2_O, NH_3_, CO, and ETH. The energy band offset at the MoS_2_-MoO_3_ heterojunction results in interfacial charge transfer leading to a *p*-type inversion layer in MoS_2_, and the gas sensing mechanism can be explained by the charge transfer between the analytical gas molecules and the *p*-type MoS_2_ inversion layer at the MoS_2_-MoO_3_ heterojunction. The efficient sensing performance for NO_2_ can be attributed to the enhanced affinity between the paramagnetic NO_2_ molecules and the ferromagnetic sensing layer. This work demonstrates a promising gas-sensing platform based on MoS_2_@MoO_3_ heterostructures and could also have implications for broader applications that utilize the unique electronic and magnetic properties of hybrid 2D metal-oxide/TMDCs heterostructures.

## Data Availability

Not applicable.

## Supplementary Materials

The following supporting information can be downloaded at: https://www.mdpi.com/article/10.3390/nano12081303/s1, Figure S1. XPS wide survey spectrum of sulfurized MoO_3_, Figure S2. Schematic illustration for the multi-channel gas-sensing characterization system. Inset shows an optical image of the MoS_2_@MoO_3_-based gas sensors. Figure S3. Transient resistance of MoS2@MoO_3_-based gas sensor towards NO_2_ in range of 0.5 ppm to 10 ppm. Figure S4. Response (0–90%) and recovery time (100–10%) of the MoS_2_@MoO_3_-based sensor to 10 ppm NO_2_ at 125 °C. Figure S5. Long-term stability of MoS_2_@MoO_3_ sensors towards 10 ppm NO_2_ under the operating temperature of 125 °C over a period of 56 days.

## References

[1] Bernstein J.A., Alexis N., Barnes C., Bernstein I.L., Nel A., Peden D., Diaz-Sanchez D., Tarlo S.M., Williams P.B., Bernstein J.A. (2004). Health effects of air pollution. J. Allergy Clin. Immunol..

[2] Bernstein J.A., Alexis N., Bacchus H., Bernstein I.L., Fritz P., Horner E., Li N., Mason S., Nel A., Oullette J. (2008). The health effects of nonindustrial indoor air pollution. J. Allergy Clin. Immunol..

[3] Das A., Dost R., Richardson T., Grell M., Morrison J.J., Turner M.L. (2007). A Nitrogen Dioxide Sensor Based on an Organic Transistor Constructed from Amorphous Semiconducting Polymers. Adv. Mater..

[4] Fernández-Sánchez J.F., Fernández I., Steiger R., Beer R., Cannas R., Spichiger-Keller U.E. (2007). Second Generation Nanostructured Metal Oxide Matrices to Increase the Thermal Stability of CO and NO_2_ Sensing Layers Based on Iron(II) Phthalocyanine. Adv. Funct. Mater..

[5] Donarelli M., Prezioso S., Perrozzi F., Bisti F., Nardone M., Giancaterini L., Cantalini C., Ottaviano L. (2015). Response to NO_2_ and other gases of resistive chemically exfoliated MoS_2_-based gas sensors. Sens. Actuator B Chem..

[6] Ji S., Wang H., Wang T., Yan D. (2013). A High-Performance Room-Temperature NO_2_ Sensor Based on An Ultrathin Heterojunction Film. Adv. Mater..

[7] Kumar S., Pavelyev V., Mishra P., Tripathi N., Sharma P., Calle F. (2020). A review on 2D transition metal di-chalcogenides and metal oxide nanostructures based NO_2_ gas sensors. Mater. Sci. Semicond. Process..

[8] Korotcenkov G., Brinzari V., Cho B.K. (2016). Conductometric gas sensors based on metal oxides modified with gold nanoparticles: A review. Microchim. Acta.

[9] Geeta Rani B., Saisri R., Kailasa S., Sai Bhargava Reddy M., Maseed H., Venkateswara Rao K. (2020). Architectural tailoring of orthorhombic MoO_3_ nanostructures toward efficient NO_2_ gas sensing. J. Mater. Sci..

[10] Rahman F., Zavabeti A., Rahman M.A., Arash A., Mazumder A., Walia S., Sriram S., Bhaskaran M., Balendhran S. (2019). Dual Selective Gas Sensing Characteristics of 2D α-MoO_3−x_ via a Facile Transfer Process. ACS Appl. Mater. Interfaces.

[11] Yang S.X., Jiang C.B., Wei S.H. (2017). Gas sensing in 2D materials. Appl. Phys. Rev..

[12] Di Bartolomeo A. (2020). Emerging 2D Materials and Their Van Der Waals Heterostructures. Nanomaterials.

[13] Joshi N., Hayasaka T., Liu Y., Liu H., Oliveira O.N., Lin L. (2018). A review on chemiresistive room temperature gas sensors based on metal oxide nanostructures, graphene and 2D transition metal dichalcogenides. Microchim. Acta.

[14] Li Z., Qiao H., Guo Z., Ren X., Huang Z., Qi X., Dhanabalan S.C., Ponraj J.S., Zhang D., Li J. (2018). High-Performance Photo-Electrochemical Photodetector Based on Liquid-Exfoliated Few-Layered InSe Nanosheets with Enhanced Stability. Adv. Funct. Mater..

[15] Zhang L., Khan K., Zou J., Zhang H., Li Y. (2019). Recent Advances in Emerging 2D Material-Based Gas Sensors: Potential in Disease Diagnosis. Adv. Mater. Interfaces.

[16] Donarelli M., Ottaviano L. (2018). 2D Materials for Gas Sensing Applications: A Review on Graphene Oxide, MoS_2_, WS_2_ and Phosphorene. Sensors.

[17] Novoselov K.S., Mishchenko A., Carvalho A., Castro Neto A.H. (2016). 2D materials and van der Waals heterostructures. Science.

[18] Cao R., Zhou B., Jia C., Zhang X., Jiang Z. (2015). Theoretical study of the NO, NO_2_, CO, SO_2_, and NH_3_ adsorptions on multi-diameter single-wall MoS_2_ nanotube. J. Phys. D Appl. Phys..

[19] Bermudez V.M. (2020). Computational Study of the Adsorption of NO_2_ on Monolayer MoS_2_. J. Phys. Chem. C.

[20] Zheng W., Xu Y., Zheng L., Yang C., Pinna N., Liu X., Zhang J. (2020). MoS_2_ Van der Waals p–n Junctions Enabling Highly Selective Room-Temperature NO_2_ Sensor. Adv. Funct. Mater..

[21] Hau H.H., Duong T.T.H., Man N.K., Thi Viet Nga T., Thi Xuan C., Thi Thanh Le D., Van Toan N., Hung C.M., Van Duy N., Van Hieu N. (2021). Enhanced NO_2_ gas-sensing performance at room temperature using exfoliated MoS_2_ nanosheets. Sens. Actuators A Phys..

[22] Kumbhakar P., Chowde Gowda C., Mahapatra P.L., Mukherjee M., Malviya K.D., Chaker M., Chandra A., Lahiri B., Ajayan P.M., Jariwala D. (2021). Emerging 2D metal oxides and their applications. Mater. Today.

[23] Wu D., Qi D., Liu J., Wang Z., Hao Q., Hong G., Liu F., Ouyang F., Zhang W. (2021). Growth of centimeter-scale single crystal MoO_3_ ribbons for high performance ultraviolet photodetectors. Appl. Phys. Lett..

[24] Duraisamy S., Ganguly A., Sharma P.K., Benson J., Davis J., Papakonstantinou P. (2021). One-Step Hydrothermal Synthesis of Phase-Engineered MoS_2_/MoO_3_ Electrocatalysts for Hydrogen Evolution Reaction. ACS Appl. Nano Mater..

[25] He R., Lai H., Wang S., Chen T., Xie F., Chen Q., Liu P., Chen J., Xie W. (2020). Few-layered vdW MoO_3_ for sensitive, uniform and stable SERS applications. Appl. Surf. Sci..

[26] Du Y., He J., Hou G., Yuan F. (2020). α-MoO_3_ sheets with high exposed plane reinforced by thermal plasma for stable Li-ion storage. Electrochim. Acta.

[27] Li W., Xing K., Liu P., Chuang C., Lu Y.-R., Chan T.-S., Tesfamichael T., Motta N., Qi D.-C. (2021). Ultrasensitive NO_2_ Gas Sensors Based on Layered α-MoO_3_ Nanoribbons. Adv. Mater. Technol..

[28] Balendhran S., Walia S., Nili H., Ou J.Z., Zhuiykov S., Kaner R.B., Sriram S., Bhaskaran M., Kalantar-zadeh K. (2013). Two-Dimensional Molybdenum Trioxide and Dichalcogenides. Adv. Funct. Mater..

[29] Malik R., Joshi N., Tomer V.K. (2021). Advances in the designs and mechanisms of MoO_3_ nanostructures for gas sensors: A holistic review. Mater. Adv..

[30] Wei Z., Hai Z., Akbari M.K., Qi D., Xing K., Zhao Q., Verpoort F., Hu J., Hyde L., Zhuiykov S. (2018). Atomic layer deposition-developed two-dimensional α-MoO_3_ windows excellent hydrogen peroxide electrochemical sensing capabilities. Sens. Actuators B Chem..

[31] Li W., Ou Q., Wang X., Xing K., Tesfamichael T., Motta N., Qi D.-C. (2022). Large-sized α-MoO_3_ layered single crystals for superior NO_2_ gas sensing. Appl. Surf. Sci..

[32] Mei J., Liao T., Sun Z. (2021). 2D/2D Heterostructures: Rational Design for Advanced Batteries and Electrocatalysis. Energy Environ. Mater..

[33] Bag A., Lee N.-E. (2019). Gas sensing with heterostructures based on two-dimensional nanostructured materials: A review. J. Mater. Chem. C.

[34] Guo S., Li X., Ren X., Yang L., Zhu J., Wei B. (2018). Optical and Electrical Enhancement of Hydrogen Evolution by MoS_2_@MoO_3_ Core–Shell Nanowires with Designed Tunable Plasmon Resonance. Adv. Funct. Mater..

[35] Hou X., Mensah A., Zhao M., Cai Y., Wei Q. (2020). Facile controlled synthesis of monodispersed MoO_3_-MoS_2_ hybrid nanospheres for efficient hydrogen evolution reaction. Appl. Surf. Sci..

[36] Chen Z., Cummins D., Reinecke B.N., Clark E., Sunkara M.K., Jaramillo T.F. (2011). Core-shell MoO_3_-MoS_2_ Nanowires for Hydrogen Evolution: A Functional Design for Electrocatalytic Materials. Nano Lett..

[37] Zhao S., Zha Z., Liu X., Tian H., Wu Z., Li W., Sun L.-B., Liu B., Chen Z. (2020). Core-Sheath Structured MoO_3_@MoS_2_ Composite for High-Performance Lithium-Ion Battery Anodes. Energy Fuels.

[38] Singh S., Guleria A., Singh N., Sharma S. (2021). Highly responsive room-temperature ammonia sensing properties of MoS_2_/MoO_3_ nano-composite. Mater. Today Proc..

[39] Li J., Xu X., Huang B., Lou Z., Li B. (2021). Light-Induced In Situ Formation of a Nonmetallic Plasmonic MoS_2_/MoO_3−x_ Heterostructure with Efficient Charge Transfer for CO_2_ Reduction and SERS Detection. ACS Appl. Mater. Interfaces.

[40] Pondick J.V., Woods J.M., Xing J., Zhou Y., Cha J.J. (2018). Stepwise Sulfurization from MoO_3_ to MoS_2_ via Chemical Vapor Deposition. ACS Appl. Nano Mater..

[41] Heo S.N., Ishiguro Y., Hayakawa R., Chikyow T., Wakayama Y. (2016). Perspective: Highly ordered MoS_2_ thin films grown by multi-step chemical vapor deposition process. APL Mater..

[42] Guo Y., Kang L., Song P., Zeng Q., Tang B., Yang J., Wu Y., Tian D., Xu M., Zhao W. (2021). MoO_3_–MoS_2_ vertical heterostructures synthesized via one-step CVD process for optoelectronics 2D. Materials.

[43] Weber T., Muijsers J.C., van Wolput J.H.M.C., Verhagen C.P.J., Niemantsverdriet J.W. (1996). Basic reaction steps in the sulfidation of crystalline MoO_3_ to MoS_2_, as studied by X-ray photoelectron and infrared emission spectroscopy. J. Phys. Chem..

[44] Kumar R., Goel N., Mishra M., Gupta G., Fanetti M., Valant M., Kumar M. (2018). Growth of MoS_2_–MoO_3_ Hybrid Microflowers via Controlled Vapor Transport Process for Efficient Gas Sensing at Room Temperature. Adv. Mater. Interfaces.

[45] Mohammadbeigi M., Jamilpanah L., Rahmati B., Mohseni S.M. (2019). Sulfurization of planar MoO_3_ optical crystals: Enhanced Raman response and surface porosity. Mater. Res. Bull..

[46] Liu H., Lin M., Guo S. (2021). Morphological and structural evolutions of α-MoO_3_ single crystal belts towards MoS_2_/MoO_2_ heterostructures upon post-growth thermal vapor sulfurization. Appl. Surf. Sci..

[47] Joya M.R., Alfonso J.E., Moreno L.C. (2019). Photoluminescence and Raman studies of alpha-MoO_3_ doped with erbium and neodymium. Curr. Sci..

[48] Diaz-Droguett D.E., El Far R., Fuenzalida V.M., Cabrera A.L. (2012). In situ-Raman studies on thermally induced structural changes of porous MoO_3_ prepared in vapor phase under He and H_2_. Mater. Chem. Phys..

[49] Camacho-López M.A., Escobar-Alarcón L., Picquart M., Arroyo R., Córdoba G., Haro-Poniatowski E. (2011). Micro-Raman study of the m-MoO_2_ to α-MoO_3_ transformation induced by cw-laser irradiation. Opt. Mater..

[50] Silveira J.V., Batista J.A., Saraiva G.D., Mendes Filho J., Souza Filho A.G., Hu S., Wang X. (2010). Temperature dependent behavior of single walled MoO_3_ nanotubes: A Raman spectroscopy study. Vib. Spectrosc..

[51] Singh S., Deb J., Sarkar U., Sharma S. (2021). MoS_2_/MoO_3_ Nanocomposite for Selective NH_3_ Detection in a Humid Environment. ACS Sustain. Chem. Eng..

[52] Windom B.C., Sawyer W.G., Hahn D.W. (2011). A Raman Spectroscopic Study of MoS_2_ and MoO_3_: Applications to Tribological Systems. Tribol. Lett..

[53] Bera A., Sood A.K., Wang Z.M. (2014). Insights into Vibrational and Electronic Properties of MoS_2_ Using Raman, Photoluminescence, and Transport Studies. MoS_2_: Materials, Physics, and Devices.

[54] Wang D., Li J.-N., Zhou Y., Xu D.-H., Xiong X., Peng R.-W., Wang M. (2016). Van der Waals epitaxy of ultrathin α-MoO_3_ sheets on mica substrate with single-unit-cell thickness. Appl. Phys. Lett..

[55] Song L.X., Xia J., Dang Z., Yang J., Wang L.B., Chen J. (2012). Formation, structure and physical properties of a series of α-MoO_3_ nanocrystals: From 3D to 1D and 2D. Cryst. Eng. Comm..

[56] Song S., Wang J., Peng T., Fu W., Zan L. (2018). MoS_2_-MoO_3−x_ hybrid cocatalyst for effectively enhanced H_2_ production photoactivity of AgIn_5_S_8_ nano-octahedrons. Appl. Catal. B Environ..

[57] Szoszkiewicz R., Rogala M., Dąbrowski P. (2020). Surface-Bound and Volatile Mo Oxides Produced During Oxidation of Single MoS_2_ Crystals in Air and High Relative Humidity. Materials.

[58] Hao S., Yang B., Gao Y. (2017). Chemical vapor deposition growth and characterization of drop-like MoS_2_/MoO_2_ granular films. Phys. Status Solidi.

[59] Wang L., Ji X., Wang T., Zhang Q. (2018). Novel Red Emission from MoO_3_/MoS_2_–MoO_2_–MoO_3_ Core–Shell Belt Surface. ACS Appl. Mater. Interfaces.

[60] Yoon A., Kim J.H., Yoon J., Lee Y., Lee Z. (2020). van der Waals Epitaxial Formation of Atomic Layered α-MoO_3_ on MoS_2_ by Oxidation. ACS Appl. Mater. Interfaces.

[61] Kibsgaard J., Chen Z., Reinecke B.N., Jaramillo T.F. (2012). Engineering the surface structure of MoS_2_ to preferentially expose active edge sites for electrocatalysis. Nat. Mater..

[62] Brown N.M.D., Cui N., McKinley A. (1998). An XPS study of the surface modification of natural MoS_2_ following treatment in an RF-oxygen plasma. Appl. Surf. Sci..

[63] Ou J.Z., Ge W., Carey B., Daeneke T., Rotbart A., Shan W., Wang Y., Fu Z., Chrimes A.F., Wlodarski W. (2015). Physisorption-Based Charge Transfer in Two-Dimensional SnS_2_ for Selective and Reversible NO_2_ Gas Sensing. ACS Nano.

[64] Zhou X.Y., Wang R., Meng L.J., Xu X.Y., Hu J.G., Pan J. (2018). Achieving half-metallicity in zigzag MoS_2_ nanoribbon with a sulfur vacancy by edge passivation. J. Phys. D Appl. Phys..

[65] Lee D.J., Lee Y., Kwon Y.H., Choi S.H., Yang W., Kim D.Y., Lee S. (2019). Room-Temperature Ferromagnetic Ultrathin α-MoO_3_:Te Nanoflakes. ACS Nano.

[66] Brinkman A., Huijben M., van Zalk M., Huijben J., Zeitler U., Maan J.C., van der Wiel W.G., Rijnders G., Blank D.H.A., Hilgenkamp H. (2007). Magnetic effects at the interface between non-magnetic oxides. Nat. Mater..

[67] Shidpour R., Manteghian M. (2010). A density functional study of strong local magnetism creation on MoS_2_ nanoribbon by sulfur vacancy. Nanoscale.

[68] Rusydi A., Dhar S., Barman A.R., Ariando n., Qi D.C., Motapothula M., Yi J.B., Santoso I., Feng Y.P., Yang K. (2012). Cationic-vacancy-induced room-temperature ferromagnetism in transparent, conducting anatase Ti_1−x_Ta_x_O_2_ (x∼0.05) thin films. Philos. Trans. R. Soc. A Math. Phys. Eng. Sci..

[69] Mao Y., Li W., Sun X., Ma Y., Xia J., Zhao Y., Lu X., Gan J., Liu Z., Chen J. (2012). Room-temperature ferromagnetism in hierarchically branched MoO_3_ nanostructures. Crystengcomm.

[70] Botello-Méndez A.R., López-Urías F., Terrones M., Terrones H. (2009). Metallic and ferromagnetic edges in molybdenum disulfide nanoribbons. Nanotechnology.

[71] Li Y., Song Z., Li Y., Li S., Wang H., Song B., Gao B., Du X. (2019). Synthesis of Mo_4_O_11_@MoO_3_ nanobelts and their improved sensing performance to NO_2_ gas. Mater. Res. Express.

[72] Analytical Methods C. (1987). Recommendations for the definition, estimation and use of the detection limit. Analyst.

[73] Shafiei M., Bradford J., Khan H., Piloto C., Wlodarski W., Li Y., Motta N. (2018). Low-operating temperature NO_2_ gas sensors based on hybrid two-dimensional SnS_2_-reduced graphene oxide. Appl. Surf. Sci..

[74] Acharyya D., Huang K.Y., Chattopadhyay P.P., Ho M.S., Fecht H.J., Bhattacharyya P. (2016). Hybrid 3D structures of ZnO nanoflowers and PdO nanoparticles as a highly selective methanol sensor. Analyst.

[75] Russell S.A.O., Cao L., Qi D.C., Tallaire A., Crawford K.G., Wee A.T.S., Moran D.A.J. (2013). Surface transfer doping of diamond by MoO_3_: A combined spectroscopic and Hall measurement study. Appl. Phys. Lett..

[76] Li S., Ma Y., Ouedraogo N.A.N., Liu F., You C., Deng W., Zhang Y. (2022). p-/n-Type modulation of 2D transition metal dichalcogenides for electronic and optoelectronic devices. Nano Res..

[77] Miller D.R., Akbar S.A., Morris P.A. (2014). Nanoscale metal oxide-based heterojunctions for gas sensing: A review. Sens. Actuator B-Chem..

[78] Xing K., Xiang Y., Jiang M., Creedon D.L., Akhgar G., Yianni S.A., Xiao H., Ley L., Stacey A., McCallum J.C. (2020). MoO_3_ induces p-type surface conductivity by surface transfer doping in diamond. Appl. Surf. Sci..

[79] Xing K., Li W., Della Gaspera E., van Embden J., Zhang L., Yianni S.A., Creedon D.L., Wang T., McCallum J.C., Wang L. (2021). Surface transfer doping of diamond using solution-processed molybdenum trioxide. Carbon.

[80] Keyshar K., Berg M., Zhang X., Vajtai R., Gupta G., Chan C.K., Beechem T.E., Ajayan P.M., Mohite A.D., Ohta T. (2017). Experimental Determination of the Ionization Energies of MoSe_2_, WS_2_, and MoS_2_ on SiO_2_ Using Photoemission Electron Microscopy. ACS Nano.

[81] McDonnell S., Azcatl A., Addou R., Gong C., Battaglia C., Chuang S., Cho K., Javey A., Wallace R.M. (2014). Hole Contacts on Transition Metal Dichalcogenides: Interface Chemistry and Band Alignments. ACS Nano.

[82] Pal S., Mukherjee S., Nand M., Srivastava H., Mukherjee C., Jha S.N., Ray S.K. (2020). Si compatible MoO_3_/MoS_2_ core-shell quantum dots for wavelength tunable photodetection in wide visible range. Appl. Surf. Sci..

[83] Hung C.M., Vuong V.A., Duy N.V., An D.V., Hieu N.V., Kashif M., Hoa N.D. (2020). Controlled Growth of Vertically Oriented Trilayer MoS_2_ Nanoflakes for Room-Temperature NO_2_ Gas Sensor Applications. Phys. Status Solidi.

[84] Yan W., Yan W., Chen T., Xu J., Tian Q., Ho D. (2020). Size-Tunable Flowerlike MoS_2_ Nanospheres Combined with Laser-Induced Graphene Electrodes for NO_2_ Sensing. ACS Appl. Nano Mater..

[85] Thang N.T., Hong L.T., Thoan N.H., Hung C.M., Van Duy N., Van Hieu N., Hoa N.D. (2020). Controlled synthesis of ultrathin MoS_2_ nanoflowers for highly enhanced NO_2_ sensing at room temperature. RSC Adv..

[86] Kwon H.I. Low-temperature-operated sensitive NO_2_ gas sensors based on p-type SnO thin-film and thin-film transistors. Proceedings of the 2020 27th International Workshop on Active-Matrix Flatpanel Displays and Devices (AM-FPD).

[87] Yu L., Guo F., Liu S., Qi J., Yin M., Yang B., Liu Z., Fan X.H. (2016). Hierarchical 3D flower-like MoS_2_ spheres: Post-thermal treatment in vacuum and their NO_2_ sensing properties. Mater. Lett..

[88] Zhu Q., Wang H., Yang J., Xie C., Zeng D., Zhao N. (2018). Red Phosphorus: An Elementary Semiconductor for Room-Temperature NO_2_ Gas Sensing. ACS Sens..

[89] Yamada Y., Ogita M. (2003). Transient response of resistive-type NO_2_ sensor on temperature change. Sens. Actuators B Chem..

[90] Yang L., Xie C., Zhang G., Zhao J., Yu X., Zeng D., Zhang S. (2014). Enhanced response to NO_2_ with CuO/ZnO laminated heterostructured configuration. Sens. Actuators B Chem..

