# Supplementary material for: Highly Sensitive NO2 Gas Sensors Based on MoS2@MoO3 Magnetic Heterostructure
Source: Nanomaterials (Basel). 2022 Apr 11;12(8):1303. doi: 10.3390/nano12081303 (PMC9027905; doi:10.3390/nano12081303)
Supplement: Supplementary file 1 [file nanomaterials-12-01303-s001.zip › nanomaterials-1610630-supplementary.pdf]

nanomaterials

MDPI

Supplementary Material

Highly Sensitive NO_2 Gas Sensors Based on MoS_2@MoO_3 Magnetic Heterostructure

Wei Li [1,2], Mahboobeh Shahbazi [1], Kaijian Xing [3], Tuquabo Tesfamichael [2,4,*], Nunzio Motta [1,2,*] and Dong-Chen Qi [1,2,*]

[1] School of Chemistry and Physics, Queensland University of Technology, Brisbane, QLD 4001, Australia; w73.li@hdr.qut.edu.au (W.L.); mahboobeh.shahbazi@qut.edu.au (M.S.)
[2] Centre for Materials Science, Queensland University of Technology, Brisbane, QLD 4001, Australia
[3] School of Physics and Astronomy, Monash University, Clayton, VIC 3800, Australia; kaijian.xing@monash.edu
[4] School of Mechanical, Medical and Process Engineering, Queensland University of Technology, Brisbane, QLD 4001, Australia
* Correspondence: t.tesfamichael@qut.edu.au (T.T.); n.motta@qut.edu.au (N.M.); dongchen.qi@qut.edu.au (D.-C.Q.)

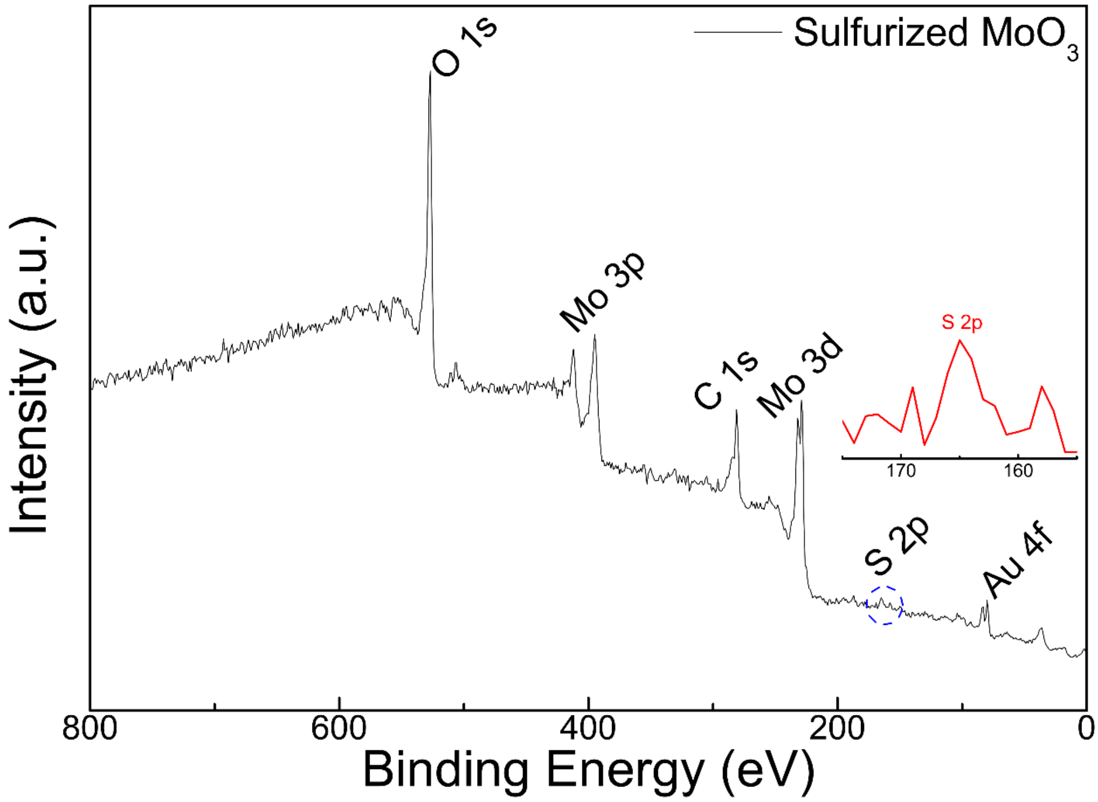

Figure S1. XPS wide survey spectrum of sulfurized MoO_3.

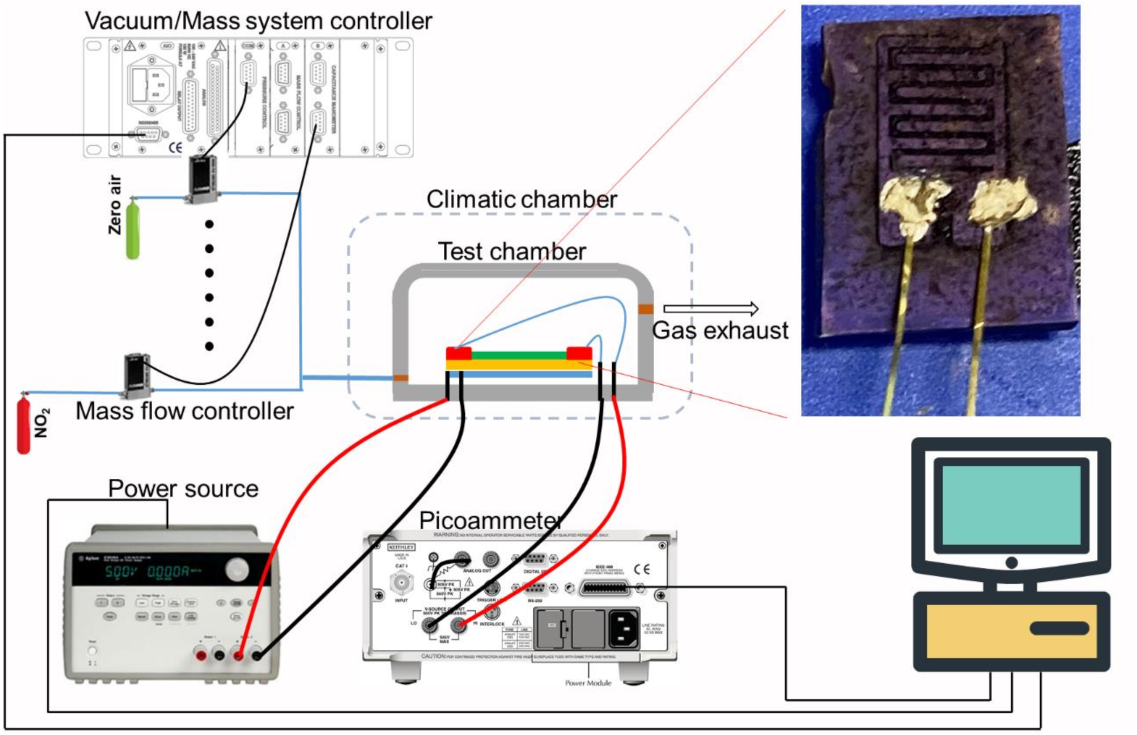

Figure S2. Schematic illustration for the multi-channel gas sensing characterization system. Inset shows an optical image of the MoS_2@MoO_3 based gas sensors.

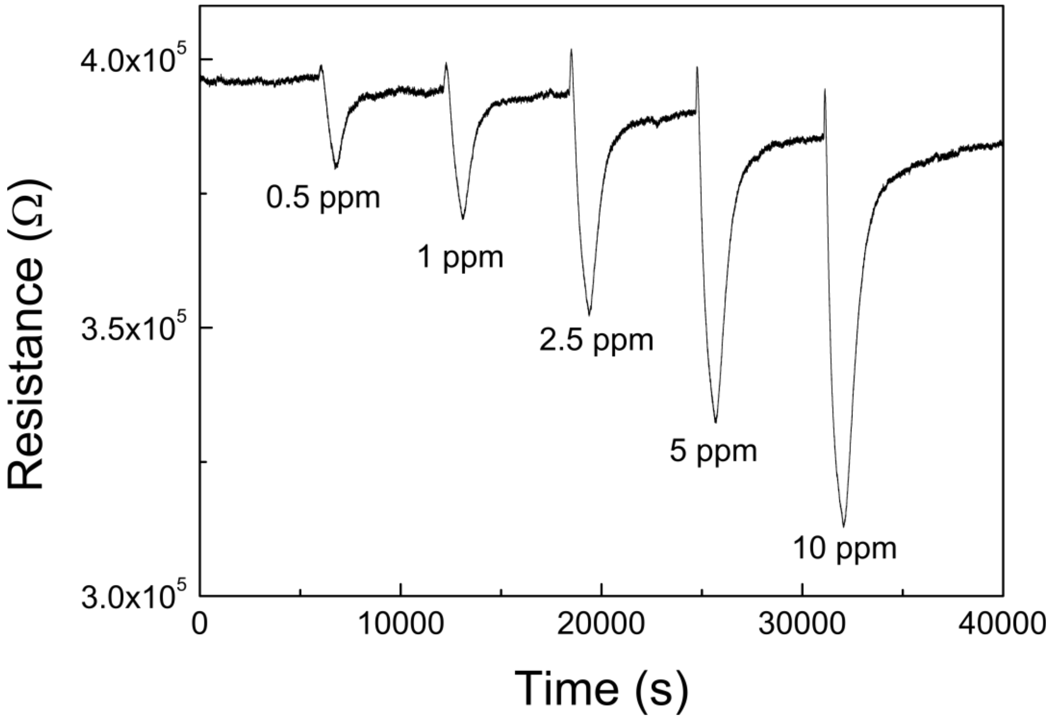

Figure S3. Transient resistance of MoS_2@MoO_3 based gas sensor towards NO_2 in range of 0.5 ppm to 10 ppm.

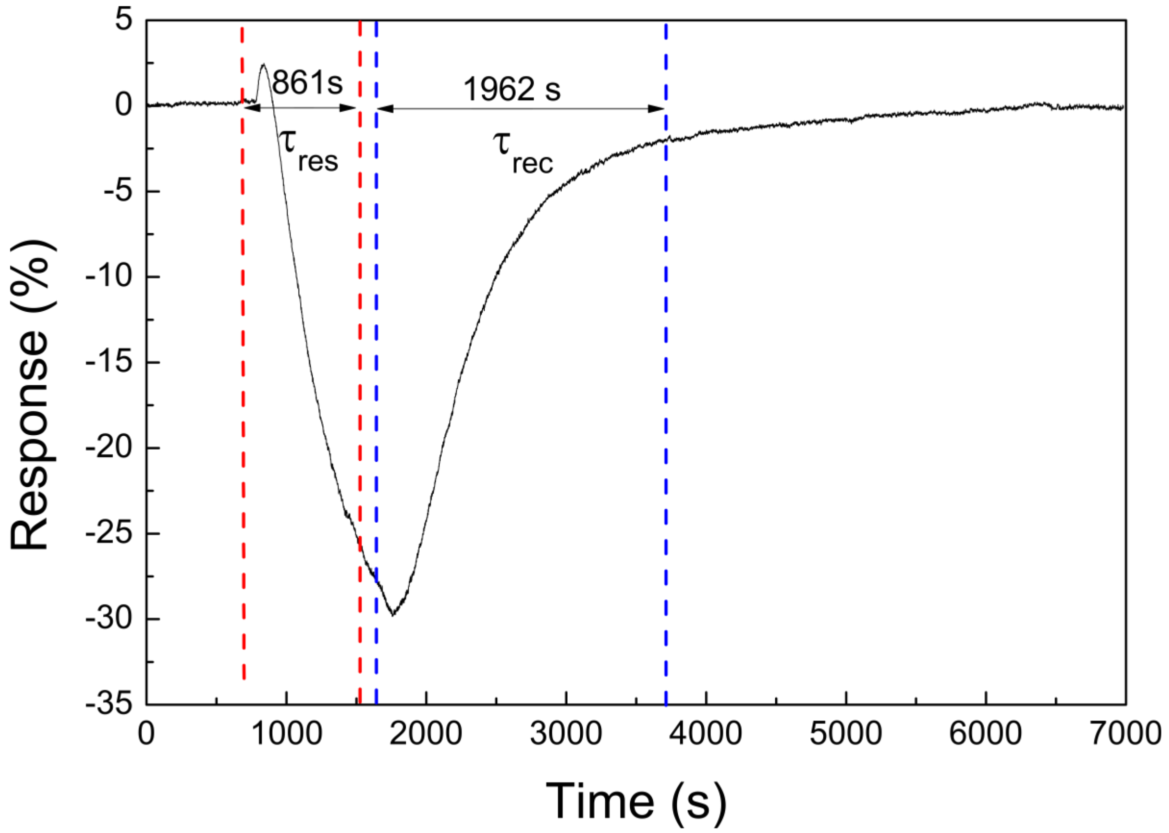

Figure S4. Response (0-90%) and recovery time (100-10%) of the MoS_2@MoO_3 based sensor to 10 ppm NO_2 at 125 °C.

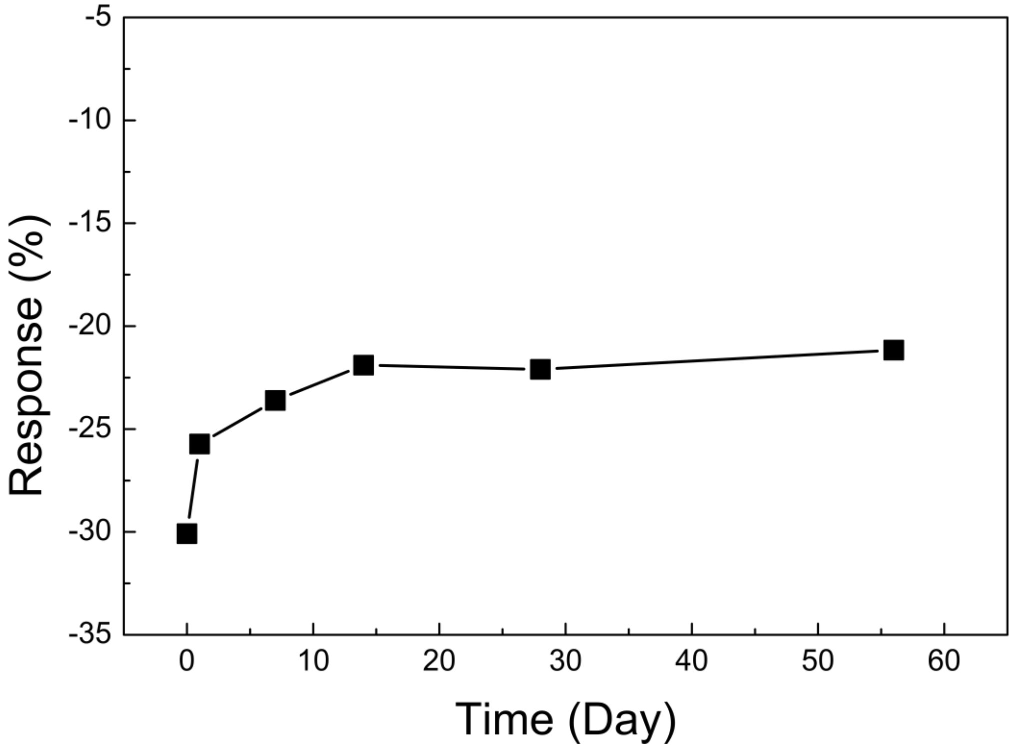

Figure S5. Long-term stability of MoS_2@MoO_3 sensors towards 10 ppm NO_2 under the operating temperature of 125 °C over a period of 56 days.
